# Education, Income, and Happiness: Evidence From China

**DOI:** 10.3389/fpubh.2022.855327

**Published:** 2022-04-12

**Authors:** Dongliang Yang, Ge Zheng, Haoran Wang, Mingna Li

**Affiliations:** ^1^Northeast Asian Research Center, Jilin University, Changchun, China; ^2^Department of Regional Economics, School of Northeast Asian, Jilin University, Changchun, China; ^3^Department of Chinese as a Foreign Language, College of Literature, Changchun University, Changchun, China

**Keywords:** happiness, education, income, migrant population, China

## Abstract

Happiness is the continuous joy that people experience when they are satisfied with their lives long term, and is the ultimate goal pursued by all citizens. In this study, we investigate the relationship between education, income, and happiness in the migrant population in China. Using 1,31,186 individuals in the 2012 China Migrants Dynamic Survey (CMDS) as research samples, the estimated results of ordinal logistic regression show that education, including secondary education and higher education, has a significant and direct impact on individual happiness, and that the impact of education on happiness can also be mediated by income as an intermediary mechanism. In addition, factors such as gender, flow distance, flow time, employment status, type of housing, number of children, degree of preference for the city, and degree of discrimination by locals have obvious effects on happiness. This work provides important insights for countries seeking to implement an active education policy in order to increase economic income and thus achieve the development goal of universal happiness among their citizens.

## Introduction

Happiness is the most direct response of people to life satisfaction and a positive psychological state of happiness. As an important indicator of public health, it is increasingly becoming the focus of health and economic policies. In 2010, the National Bureau of Statistics of the United Kingdom designed the index of happiness and studied national citizen's happiness. In 2011, the German government established the Economic Growth, Happiness and Quality of Life Research Committee to research the German Happiness. In 2021, United Nations evaluated the impact of the COVID-19 pandemic and the response measures of various countries, and published the “2021 World Happiness Index Report” based on factors such as health, work pressure, social support, freedom, and income. Among the 149 countries surveyed in the world, China ranked 84th with a score of 5.339 (1). However, compared with the ranking of China's economy in the world, China's happiness index ranked relatively low.

Happiness is a complex concept involving multiple disciplines such as neurophysiology, psychology, and economics. Happiness is a neurophysiological process produced by comprehensive coordination of the prefrontal cortex, cingulate cortex, and amygdale (2). It is composed of mental activity that consists of two basic levels of cognition and emotion, which in turn involve three stages: emotional response, emotional experience, and cognitive evaluation (3, 4). Emotional response and emotional experience are the individual real-time reaction to the objective circumstances and state of life (5, 6). Cognitive evaluation emphasizes the rational or intellectual evaluation of the individual expectations of happiness and real-life satisfaction, which is a positive psychological experience based on life satisfaction (7). Happiness is the continuous happy mood that people have when they are in a state of life satisfaction long term. Whether they are happy depends on their balance between happiness and painful emotional experiences (8). From the economic perspective, happiness is closer to the concept of utility, wherein, as proposed by Jevons, the satisfying effects of consumer goods are applied to determine the level of welfare from the perspective of utility (9).

Happiness is closely related to education, which is often portrayed as one of the key (direct or indirect) aims of education (10, 11). Previous studies have shown that education can change an individual's cognitive abilities, and the higher the level of education, the happier the individual (12, 13). Being educated gives individuals have increased control over their work and life, which helps to alleviate psychological worries of being controlled and deprived: this is also conducive to dealing with problems more actively and optimistically and thus to achieve self-realization and independence in work (14, 15). Meanwhile, the empirical research on education and happiness indicates that after controlling for the influence of variables such as health, income, and occupation, education has a significantly positive impact on happiness. Moussa and Ali (16) measured the level of happiness of higher education students and their relationship to academic success during the COVID-19, and data analysis showed that there was a positive correlation between happiness and academic achievement levels for students in higher education in the UAE. A recent study by Nikolaev (17) used the 2001–2013 Household Income and Labour Dynamics in Australia (HILDA) survey data to examine the relationship between higher education and three different measures of happiness; the results showed that the higher the level of education of individuals, the more satisfied they were with most areas of life (finance, employment opportunities, neighborhood, local community, children at home) and the higher their level of happiness. From a long-term development perspective, people with a higher education level can achieve their expectations and goals more effectively, and therefore experience more control over their lives and undoubtedly be happier (18–20).

Through education, not only can individuals' cognitive abilities be improved, but also their knowledge and skills, thus enabling more economic value and a higher level of consumer utility to be obtained. The accumulation of knowledge through education is the main way for individuals to improve their human capital; this human capital enables them to increase their income by improving their individual cognitive ability and work efficiency, that is, “education-human capital-income” (21). Under the free competitive market conditions, educational qualifications are viewed as indicators of highly skilled labor (22). Higher education diplomas signify that the individual has higher skills, which translate into higher remuneration (23). Generally, individuals with higher educational backgrounds earn higher wages (24, 25). Furthermore, high income can expand the set of feasible choices available to consumers, allow consumers to meet their individual preferences and needs better to improve the level of utility, thus, providing them a higher level of happiness as individuals (26). After Samuelson proposed the consumption revealed preference theory (27), the Marshall's demand function and the indirect utility function were used to correlate utility with income through Roy's Identity, thus achieving the “income-consumption-utility-happiness” conversion. Examining the relationship between education and happiness, Tran et al. (28) explored the mechanism underlying the impact of education on women's happiness in Australia when income is introduced as a mediating variable into the regression of education on happiness; the findings showed the coefficient of education's influence on happiness dropped significantly and the mediating effect of income on happiness was still statistically significant, indicating that income is one of the important mediators for education to have a positive impact on happiness. Even within families, education disparities can affect couples' happiness, and income remains a channel for couples to achieve happiness (29). Therefore, education may increase the individual perception of happiness by increasing the individual's economic income.

However, there is a complex relationship between education and happiness, wherein happiness is mainly determined by the gap between actual income and expected life satisfaction; people with higher education levels also have higher expected goals (30). Although individuals with higher education receive higher income, they are also more likely to find that their income expectations are not met, and the frustration of such individual expectations will have a negative impact on happiness (31, 32). Jongbloed (33) focused on the impact of higher education on the happiness of Europeans and found that education has little effect on satisfaction indicators such as personal optimism and self-esteem; people with higher levels of education are less likely to perceive a sense of accomplishment from their work, indicating that, higher education may not fulfill the role of improving happiness in Europe.

Furthermore, Easterlin et al. (34) found that in a given country, rich people are happier than the poor: happiness does not increase as a country's income rises over the long term, and analysis of the relationship between income and happiness revealed the level of national happiness in poor and rich countries to be almost the same, which is defined as “Easterlin paradox,” that is, there is no obvious positive correlation between income and happiness. FitzRoy and Nolan (35) studied the relationship between education, income, and happiness based on the 1996–2009 British Household Panel Survey (BHPS) and found that individuals with lower levels of education, as their income levels increase, their sense of happiness in life declines, which was consistent with the “Easterlin Paradox.” This showed that the pursuit of economic income may not bring happiness, and the pursuit of a sense of mission was the main channel for education to improve happiness (36, 37). Using internationally comparable data on more than 48,000 individuals in 24 countries, Araki (38) found a positive link between educational attainment and happiness, a relationship that was no longer substantiated once a fairer distribution of pay in the labor market was taken into account. Therefore, as discussed above, most of the evidence for this relationship to date has been limited to developed countries.

In contrast, for developing countries like China, the evidence for the link between education and happiness is still scant due to a lack of reliable data. Yang et al. (39) research believes that the happiness level of Chinese residents has doubled from 2003 to 2015, and the main factor for the improvement of happiness is the increase of income or education level. Zhang and Liu (40) used the cross-sectional data of the 2010 China Comprehensive Social Survey (CGSS) to find that the level of happiness of Chinese people with high education is higher than that of people with low education. In addition, individuals will try to compare living standards with their peers, and higher income and wealth means more material happiness (41). However, while China's economy is growing rapidly, income inequality has also increased significantly, and happiness increases with the increase in income inequality (42). At present, China is in an emerging economy transitioning to a market economy and has huge research space. By clarifying the relationship between education, income and happiness of Chinese residents, it provides a detailed reference for promoting the happiness development of Chinese residents and the development of people's livelihood and happiness in other developing countries.

In this context, personal happiness may not be related only to education itself, but also to the increase in economic income brought by education. Empirical testing of the relationship between happiness and education in the Chinese population is an important way to judge the happiness level of the Chinese population. This paper makes contributions to several aspects. First, we establish the mechanism of happiness affected by education. There is a logical relationship between education and happiness, which appears as “Education-Human capital-Income-Consumption-Utility- Happiness” transformed in turn. We use a mediation model to verify the transmission mechanism through which education affects happiness via economic income. Second, we analyze overall performance of happiness of Chinese residents, and use ordered logistic regression to test the causal effect of education on individual happiness, which provided support for the positive relationship between education and happiness. Third, there are some interesting findings of other influence factors on happiness of Chinese residents, such as individual characteristics, psychological characteristics and job characteristics have a significant impact on happiness.

Using data from the 2012 China Migrants Dynamic Survey, we find that individuals' education level is positively correlated with happiness. Education can affect individual happiness both directly and via the intermediary mechanism of income factors. That is to say, the higher the education level of an individual, the higher his/her income level and the stronger his/her sense of happiness.

The rest of the paper is organized into five sections. Section Data and Variables describe the data source and variable definitions. Section Models and Methodology describes the models and methods for the assessment of education, income, and happiness. Section Results describes the results. Section Discussion describes robustness check and Section Conclusion presents the findings.

## Data and Variables

### Data

In this study, we use data from the China Migrants Dynamic Survey (CMDS) conducted by the China Population and Family Planning Commission in 2012. The CMDS collects information on the migrant population, including information on basic demographic characteristics, family, employment, income, medical and health services, public services, health, and life experience from a representative sample of the floating population aged 15–59 years old in 31 provinces (autonomous regions and municipalities directly under the Central Government) and the Xinjiang Production and Construction Corps.

### Variable Definitions

The dependent variable in this study is self-evaluated happiness. The CMDS contains the question “do you feel happy now compared with how you felt in your hometown (place of outflow)” and provides five responses, where 1 means “very happy,” 2 means “happy,” 3 means “so-so,” 4 means “unhappy,” and 5 means “very unhappy.” We grouped these responses into an ordered scale of happiness by coding responses in reverse order. This variable is measured by 5 levels, where 1 is very unhappy and 5 is very happy.

The independent variable is education level. Considering that China's nine-year compulsory education policy covers all regions of the country, we divided education levels into elementary education, secondary education, and higher education by coding respondents who have attended elementary school, junior high school, or have not attended school as elementary education or 0; those who have attended high school or technical secondary education as secondary education or 1; and those who have attended college, undergraduate, or postgraduate education as higher education or 2.

The intermediary variable is income. Wage income includes personal wages, bonuses, overtime pay, allowances, and funds equivalent to the food and housing of work. If the surveyed person had not received their salary for the current month, the most recent salary was recorded.

Based on the review of the literature on happiness research by Easterlin and other scholars, the control variables mainly include personal characteristics, flow characteristics, social characteristics, and psychological characteristics. Personal characteristics include gender, age, household registration. There are two main variables to represent flow characteristics: flow distance and flow time. Flow distance includes inter-province flow, intra-province flow, intra-city flow. The flow time refers to the most recent date on which the respondent arrived in the city, district, or county to commence work and residence. Social characteristics include employment status and the type of housing. Employment status includes whether the individuals are employees, employers, or self-employed workers. Respondents' housing includes low-rent housing, self-rented housing, free beds in the factory, and self-owned housing. Low-rent housing refers to the respondents' housing as rented units or employer's housing; low-rent housing to that provided by the government; self-leased housing includes rented private houses and other informal residences; free beds in the factory include free housing and employment places provided by the unit or employer; self-owned houses include self-purchased houses, self-built houses, and borrowed houses. In terms of reflecting psychological factors, indicators of the adaptation and identification of the floating population to the city, such as the degree to which individuals like the city and the evaluation of whether they accept or discriminate against the locals, were selected.

The factors affecting personal income mainly include personal characteristics and job characteristics. Personal characteristics include education level, gender, age, household registration, flow distance, and flow time. Job characteristics include occupational type, industry, nature of the work, employment status, and work intensity. To classify occupations, we categorized the heads of state agencies, parties, and mass organizations, enterprises and institutions, professional and technical personnel, civil servants and related personnel, and business as the leading technical class; vendors or those employed in catering, cleaning, security, decoration, transportation, other commercial service personnel, no fixed occupations, and others were classified under the service class; workers in agriculture, forestry, animal husbandry, fishery, and water conservancy production, production, construction, and other transportation equipment operators and related personnel were classified as the production class. Categories based on the nature of the unit were as follows: public-owned units, private-owned units and foreign-funded units. The division of industries is as follows: manufacturing, electricity, coal and water production and supply, mining, construction, agriculture, forestry, animal husbandry and fishery in the statistical survey as manufacturing; wholesale and retail, accommodation and catering, and others as the sale of food and accommodation services; social services, financial and insurance real estate, transportation, warehousing and communications are classified as housing money transportation services; health, sports and social welfare, education and culture, radio, film and television, scientific research and technical services, party and government agencies and social organizations are classified as science, education, culture, and health. In terms of employment status, domestic helpers were categorized into self-employed workers, and there were three categories with self-employed workers, employees and employers. Work intensity includes daily working hours and weekly working days.

### Descriptive Statistics

Table 1 shows descriptive statistics. After excluding outliers and missing values, the sample included 1,31,186 individuals. The average happiness score is 3.74. The percentages of individuals whose answers were “very happy, happy, so-so, unhappy, and very unhappy” were 18,666 (14.23%), 62,222 (47.43%), 48,083 (36.65), 1,967 (1.50%) and 248 (0.19%), respectively. The average education level is 0.411. The numbers of individuals with elementary, secondary, and higher levels of education were 90,118, 28,186, and 12,882, respectively. The average income level is 3,128 yuan.

**Table 1:** Descriptive statistics.

**Variables**	**Definitions**	**Mean**	**Std. Dev**.	**Min**	**Max**
Happiness	1 = very unhappy, 2 = unhappy, 3 = so-so, 4 = happy, 5 = very happy	3.740	0.720	1	5
Education level	0 = elementary, 1 = secondary, 2 = higher	0.411	0.662	0	2
Income	Monthly income (yuan)	3128.048	3201.859	100	98000
Gender	1 = female, 0 = male	0.408	0.491	0	1
Age	Respondent's age	33.869	9.052	15	60
Household registration	1 = non-agricultural, 0 = agricultural	0.158	0.365	0	1
Flow distance	0 = Inter-province, 1 = Intra-province, 2 = Intra-city	0.575	0.739	0	2
Flow time	The length of the last visit to this city/district/county	4.399	4.636	0	51
Employment status	0 = employees, 1 = employers, 2 = self-employed workers	0.721	0.904	0	2
Type of housing	0 = Self-rented housing, 1 = Low-rent housing, 2 = Free beds in the factory, 3 = Owned housing	0.816	1.151	0	3
Number of children	Number of children owned by the respondent	1.380	0.716	0	7
Like the city	0 = completely disagree, 1 = disagree, 2 = basically agree, 3 = completely agree	2.438	0.558	0	3
Feelings of discrimination	0 = completely disagree, 1 = disagree, 2 = basically agree, 3 = completely agree	1.003	0.787	0	3
Industry	0 = science, education, culture and health, 1 = industry, 2 = sale, food and accommodation service, 3 = housing money transportation service	1.731	0.773	0	3
Occupational type	0 = production class, 1 = leadership class, 2 = service class	1.194	0.836	0	2
Nature of work	0 = private ownership unit, 1 = public ownership unit, 2 = foreign ownership unit	0.226	0.538	0	2
Working hours per day	The number of hours the respondent worked in a day	9.446	1.902	1	16
Working days per week	The number of days the respondent worked in a week	6.256	0.959	0.5	7

## Models and Methods

**Figure 1:**
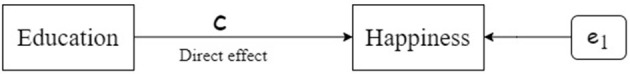
Direct effect of education on happiness.

Mediating effect analysis is an important step to test whether a variable is a mediating variable and to what extent it plays a mediating role. Figure 1 can be used to describe the relationship between education and happiness, and the path coefficient is *c*. Because the third variable is not involved, the coefficient *c* represents the total effect of education acts on happiness.

**Figure 2:**
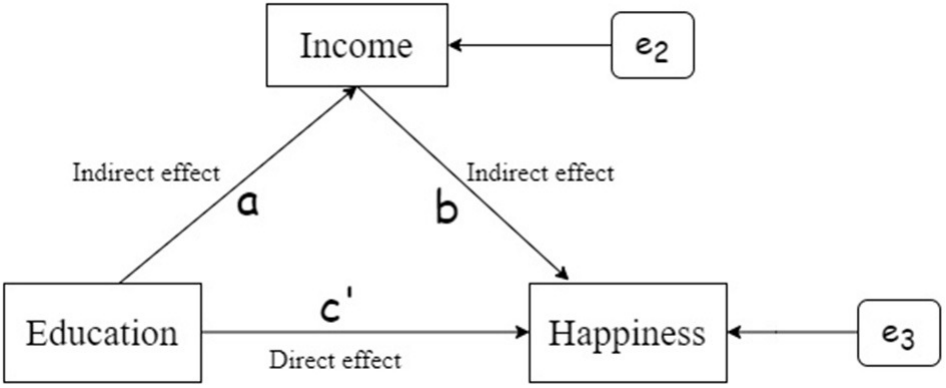
Intermediate effect of education on happiness.

Figure 2 shows the relationship between education and happiness after controlling the intermediate variable income. The coefficient *a* represents the effect of education acting on the income, and the coefficient *b* represents the effect of the intermediate variable income acting on happiness. The two constitute the indirect effect of the relationship between education and happiness. The coefficient *c*′ represents the effect of education acting on happiness after controlling the income variable, that is, the direct effect between education and happiness. Then, the total effect between variables should be equal to the direct effect plus the indirect effect: that is, the total effect equals *ab*+*c*′. Combining Figures 1, 2, we get *c* = *ab*+*c*′, where *c* is total effect, *c*′ is the direct effect, and *ab* is the intermediate effect, also called the indirect effect. The analysis of the mediation effect is to test whether the *ab* effect exists and what its proportion of the total effect is, and reflect the degree of the mediation effect.

The corresponding structural equation is:


(1) $$\begin{array}{l} \begin{array}{c} Y=cX+\text{λ}P+e_{1} \end{array} \end{array}$$

where *Y* is the dependent variable happiness; *X* is the independent variable education level; and *P* is the control variable that affects happiness, including personal characteristics, mobility characteristics, social characteristics, and psychological characteristics. The coefficient to be estimated *c* is the total effect of education on happiness, and λ is the influence of control variables on happiness.


(2) $$\begin{array}{l} M=aX+\text{λ}P\prime +\gamma W+e_{2} \end{array}$$

*M* is an intermediary variable, that is, the logarithm of individual income; *P*′ is a control variable that affects income, including personal characteristics and mobility characteristics; and *W* is a control variable reflecting work characteristics that affect individual income, including occupational type, industry, nature of work, employment status, daily working hours, and weekly working days; b the coefficient *a* to be estimated is the impact of education on income.


(3) $$\begin{array}{l} Y=c\prime X+bM+\text{λ}P+e_{3} \end{array}$$

The coefficient *c*′ to be estimated is the direct effect of education on happiness, and the coefficient *b* to be estimated is the effect of income on happiness.

Considering that the dependent variable is a group of ordinal categorical variables and the number of response variables is >2, ordinal logistic regression is suitable for the analysis of Equations (1), (3) (43). The income is a set of continuous variables, and the ordinary least square method is used for estimation. Therefore, we check the regression coefficients in three steps (44–46).

## Results

The models 1.1–1.3 in Table 2 are the estimated results for the model of the mediating effect of education on happiness. Model 1.1 is the estimated result of Equation (1) by using the ordinal logistic regression method, Pseudo R^2^ is displayed as 0.087, the LR statistic is 18795.77, and the corresponding *p* value is 0.000; therefore, the joint significance of all coefficients (except the constant term) of the entire equation is very high. Studies have found that after controlling the gender, age, flow, and psychological characteristics of the migrant population, education significantly affects happiness. Compared with elementary education, the impact of secondary education on happiness for the floating population was found to be 4.8%, passing the 1% significance test; the effect of higher education on happiness was 14.3%, which is significant at a significance level of 1%. It shows that the higher the education level of the individual, the higher their happiness.

**Table 2:** The mediating effect model estimation results of education on happiness.

	**1.1**	**1.2**	**1.3**
**Variables**	**Happiness**	**Income**	**Happiness**
Secondary education	0.048^***^	0.092^***^	0.030^*^
	(0.017)	(0.004)	(0.017)
Higher education	0.143^***^	0.297^***^	0.082^***^
	(0.027)	(0.006)	(0.028)
Income			0.176^***^
			(0.011)
Gender	0.065^***^	−0.210^***^	0.111^***^
	(0.013)	(0.003)	(0.013)
Age	−0.001	0.001^***^	0.001
	(0.001)	(0.000)	(0.001)
Household registration	−0.001	0.092^***^	−0.015
	(0.020)	(0.005)	(0.020)
Intra-province	0.185^***^	−0.130^***^	0.209^***^
	(0.014)	(0.003)	(0.015)
Intra-city	0.176^***^	−0.173^***^	0.208^***^
	(0.018)	(0.004)	(0.018)
Flow time	0.026^***^	0.001^**^	0.026^***^
	(0.001)	(0.001)	(0.001)
Employers	0.182^***^	0.409^***^	0.106^***^
	(0.019)	(0.005)	(0.020)
Self-employed workers	0.081^***^	0.133^***^	0.056^***^
	(0.014)	(0.004)	(0.014)
Type of housing	0.100^***^		0.098^***^
	(0.005)		(0.005)
Number of children	−0.019^**^		−0.017^*^
	(0.010)		(0.010)
Like the city: disagree	−1.350^***^		−1.345^***^
	(0.116)		(0.116)
Like the city: basically agree	0.275^***^		0.277^***^
	(0.100)		(0.100)
Like the city: completely agree	1.462^***^		1.469^***^
	(0.100)		(0.100)
Discriminate: disagree	−0.586^***^		−0.588^***^
	(0.015)		(0.015)
Discriminate: agree to some extent	−1.018^***^		−1.019^***^
	(0.020)		(0.020)
Discriminate: completely agree	−0.814^***^		−0.813^***^
	(0.033)		(0.033)
Manufacturing		0.049^***^	
		(0.009)	
Sale, food, and accommodation service		−0.061^***^	
		(0.008)	
Housing, money, transportation service		−0.001	
		(0.009)	
Leadership class		0.152^***^	
		(0.005)	
Service class		−0.002	
		(0.005)	
Public ownership unit		−0.080^***^	
		(0.005)	
Foreign ownership unit		0.096^***^	
		(0.007)	
Working hours per day		0.015^***^	
		(0.002)	
Working days per week		0.016^***^	
		(0.001)	
/cut1	−6.229^***^		−4.804^***^
	(0.132)		(0.160)
/cut2	−3.913^***^		−2.487^***^
	(0.108)		(0.141)
/cut3	−0.066		1.363^***^
	(0.105)		(0.139)
/cut4	2.607^***^		4.041^***^
	(0.106)		(0.140)
Constant		7.543^***^	
		(0.016)	
Observations	101527	131186	101527
Pseudo R^2^	0.087		0.088
LR chi^2^	18795.77		19043.98
Adjust R-squared		0.166	

*Robust standard errors in parentheses*;

* *p < 0.10*,

** *p < 0.05*,

*** *p < 0.01*.

Model 1.2 is the estimation result of Equation (2) obtained by using the ordinary least square method. In addition to controlling the basic personal characteristics and psychological characteristics of floating individuals, the work characteristics of individuals, such as their occupation, industry, nature of work, working hours, and other variables, are included. The results of the study show that, compared with migrant individuals in elementary education, the impact of secondary education on income is 9.2%, which is significant at the level of 1%; the impact of higher education on income is as high as 29.7%, and it is also significant at the level of 1%. It indicates an increasing trend with the level of education. Whether education is the accumulation of human capital or an indicator of high-skilled labor, individuals with higher education levels have higher income levels than those with lower levels, that is, increasing the number of years of education is still an efficient human capital investment (47).

Model 1.3 is the estimated result of Equation (3) obtained by using the ordinal logistic regression method. Pseudo R^2^ is displayed as 0.088, the LR statistic is 19043.98, and the *p* value is 0.000; therefore, the joint significance of all coefficients (except the constant term) of the entire equation is very high. After controlling the personal characteristics, flow characteristics, social characteristics, and psychological characteristics of the floating population, the individual economic income variables are introduced. It is found that the impact of income on happiness passes the 1% significance test, and the impact degree is 17.6%. It can be calculated that the mediating effects of secondary education and higher education on happiness are 1.62% (17.6% ^*^ 9.2%) and 5.23% (17.6% ^*^ 29.7%), respectively. The mediating effect among the total effect is 33.72% (17.6% ^*^ 9.2%/4.8%) and 36.55% (17.6% ^*^ 29.7%/14.3%), respectively. The results show that compared with elementary education, the direct effect of secondary education on happiness in the floating population is 3.0%, which is significant at a level of 10%. Higher education significantly effects happiness; the direct effect of 8.2% is significant at the 1% significance level. It shows that the mechanism of education on happiness is a partial mediating effect. Furthermore, education also influences happiness via the mediating factor of income, consistent with the conclusions of Ross and Willigen (48) and Chen (49).

In addition to education and income as factors that affect the happiness of migrants, the estimation results of models 1.1 and 1.3 in Table 2 show that the number of children, the distance of migrated, employment status, the degree of preference for the city, and the indicators of local discrimination significantly affect their level of happiness. For example, the larger number of children that need to be raised, the weaker their sense of happiness. Childbirth and upbringing requires personal time and money, consumes the individual's passion and energy to cope with life, affects the individual's happiness in work and life, and causes the individual's perception of happiness to decline. The influence of flow distance on happiness is significantly negative. The happiness of the intra-city and intra-province floating population is higher than that of the inter-province population. The happiness of employers and self-employed workers is significantly higher than that of the employees. Psychological factors significantly impact happiness. Happiness itself is a psychological feeling, which is inevitably closely related to other psychological states. Happiness comes from inner positive and joyful emotions. A positive attitude toward the city can obviously improve the happiness of migrants, and the evaluation of discriminatory feelings against locals is also an important reflection of such a positive attitude (50).

## Discussion

To check robustness of our results, we changed the sample range. Working hours are often an important factor that affects physical and mental health. Researchers from World Health Organization (WHO) and International Labour Organization (ILO) have conducted a global analysis of the loss of life and health caused by long working hours based on relevant data from 194 countries or regions around the world, and found that people who work 55 h or more a week compared with the standard working hours (35–40 h per week)are at higher risk of ischemic heart disease and stroke. The burden of disease caused by long hours of work accounts for about one-third of the total disease burden, and working long hours has been determined as the risk factor with the greatest burden among all occupational diseases (51). Furthermore, extended working hours will take up the free time of individuals, reduce the amount of things they like to do, and thus reduce their sense of happiness. In order to avoid the decrease in happiness caused by long working hours, we limit the number of working days to 4–6 days a week, and the daily working time is 4–12 h. According to the parameter estimation results (see Table 3) of Model 2.1, obtained by re-estimation with 57,258 individuals, compared with that of elementary education, the total effect of secondary education on happiness is 4.6% and the total effect of higher education on happiness is 10.5%. However, in Model 2.3, the direct effect of education on happiness becomes insignificant, while the impact of income on happiness is 17.2%, which is significant at a level of 1%. It can be thus inferred that secondary education and higher education have an effect on happiness, and the mediating effects were 1.75% (17.2% ^*^ 10.2%) and 5.40% (17.2% ^*^ 31.4%), respectively. The mediating effect accounted for approximately 38.14% (17.2% ^*^ 10.2%/4.6%) of the total effect, 51.44% (17.2% ^*^ 31.4%/10.5%). This finding shows that after considering the influence of working hours, the impact of education on happiness is completely mediated by the income mechanism, and education does not directly affect happiness.

**Table 3:** Regression results of restricted working time range.

	**2.1**	**2.1**	**2.3**
**Variables**	**Happiness**	**Income**	**Happiness**
Secondary education	0.046^*^	0.102^***^	0.025
	(0.026)	(0.005)	(0.026)
Higher education	0.105^***^	0.314^***^	0.037
	(0.036)	(0.007)	(0.036)
Income			0.172^***^
			(0.020)
Gender	0.062^***^	−0.225^***^	0.115^***^
	(0.020)	(0.004)	(0.021)
Age	−0.001	0.003^***^	0.001
	(0.001)	(0.001)	(0.001)
Household registration	−0.008	0.103^***^	−0.025
	(0.029)	(0.006)	(0.029)
Intra-province	0.192^***^	−0.149^***^	0.221^***^
	(0.024)	(0.005)	(0.024)
Intra-city	0.162^***^	−0.208^***^	0.200^***^
	(0.029)	(0.006)	(0.029)
Flow time	0.023^***^	0.005^***^	0.022^***^
	(0.002)	(0.001)	(0.002)
Employers	0.309^***^	0.443^***^	0.231^***^
	(0.042)	(0.010)	(0.043)
Self-employed workers	0.107^***^	0.071^***^	0.095^***^
	(0.027)	(0.006)	(0.027)
Type of Housing	0.121^***^		0.118^***^
	(0.008)		(0.008)
Number of children	−0.015		−0.014
	(0.016)		(0.016)
Like the city: disagree	−1.277^***^		−1.281^***^
	(0.183)		(0.183)
Like the city: agree to some extent	0.233		0.234
	(0.158)		(0.158)
Like the city: completely agree	1.430^***^		1.435^***^
	(0.158)		(0.158)
Discriminate: disagree	−0.550^***^		−0.555^***^
	(0.025)		(0.025)
Discriminate: agree to some extent	−0.980^***^		−0.986^***^
	(0.032)		(0.032)
Discriminate: completely agree	−0.820^***^		−0.828^***^
	(0.053)		(0.054)
Manufacturing		0.030^***^	
		(0.009)	
Sale, food, and accommodation service		−0.057^***^	
		(0.009)	
Housing, money, transportation service		0.017^*^	
		(0.009)	
Leadership class		0.159^***^	
		(0.007)	
Service class		−0.010	
		(0.006)	
Public ownership unit		−0.078^***^	
		(0.006)	
Foreign ownership unit		0.091^***^	
		(0.007)	
Working hours per day		0.012^***^	
		(0.004)	
Working days per week		0.018^***^	
		(0.001)	
/cut1	−6.184^***^		−4.796^***^
	(0.207)		(0.264)
/cut2	−3.944^***^		−2.557^***^
	(0.171)		(0.237)
/cut3	−0.087		1.304^***^
	(0.167)		(0.234)
/cut4	2.566^***^		3.961^***^
	(0.167)		(0.235)
Constant		7.502^***^	
		(0.027)	
Observations	39351	57258	39351
Pseudo R^2^	0.0855		0.0864
LR chi^2^	7206.42		7278.78
Adjust R-squared		0.236	

*Robust standard errors in parentheses*;

* *p < 0.10*,

*** *p < 0.01*.

In order to more intuitively show the marginal effect of each explanatory variable on happiness, we calculated the mean marginal effect of each explanatory variable to show that the change of the explanatory variable affects the probability of happiness taking each value. The results are shown in Table 4. When all explanatory variables are at the mean value, compared with elementary education, each level of higher education increases, the probability of an individual being very unhappy, unhappy, so-so, happy, and very happy will change at the 1% significance level by −0.0001, −0.0011, −0.0148, 0.0061, and 0.0099, respectively. The probability of an individual feeling very happy is 0.0038 higher than the probability of being happy for each level of higher education. It shows that the higher the education level of individuals, the higher the probability of obtaining happiness. This reflects that education improves people's sense of happiness and satisfaction, which is conducive to promoting people to form a good level of mental health and to participate more actively in future life.

**Table 4:** Marginal effect table of explanatory variables.

	**3.1**	**3.2**	**3.3**	**3.4**	**3.5**
**Variables**	**Very unhappy**	**Unhappy**	**So-so**	**Happy**	**Very happy**
Secondary education	−0.0001	−0.0004	−0.0054	0.0023	0.0035
Higher education	−0.0001^**^	−0.0011^**^	−0.0148^**^	0.0061^**^	0.0099^**^
income	−0.0003^***^	−0.0023^***^	−0.0319^***^	0.0136^***^	0.0208^***^
Gender	−0.0002^***^	−0.0015^***^	−0.0202^***^	0.0086^***^	0.0132^***^
Age	−0.0001	−0.0001	−0.0001	0.0001	0.0001
Household registration	0.0001	0.0002	0.0027	−0.0012	−0.0018
Intra-province	−0.0003^***^	−0.0027^***^	−0.0379^***^	0.0160^***^	0.0249^***^
Intra-city	−0.0003^***^	−0.0027^***^	−0.0378^***^	0.0160^***^	0.0248^***^
Flow time	−0.0001^***^	−0.0003^***^	−0.0048^***^	0.0021^***^	0.0031^***^
Employers	−0.0002^***^	−0.0013^***^	−0.0192^***^	0.0081^***^	0.0127^***^
Self-employed workers	−0.0001^***^	−0.0007^***^	−0.0102^***^	0.0044^***^	0.0066^***^
Type of Housing	−0.0002^***^	−0.0013^***^	−0.0178^***^	0.0076^***^	0.0116^***^
Number of children	0.0001	0.0002	0.0031	−0.0013	−0.0021
Like the city: disagree	0.0072^***^	0.0574^***^	0.2101^***^	−0.2291^***^	−0.0455^***^
Like the city: agree to some extent	−0.0006^*^	−0.0054^*^	−0.0599^**^	0.0478^**^	0.0181^**^
Like the city: completely agree	−0.0020^***^	−0.0174^***^	−0.2941^***^	0.1572^***^	0.1571^***^
Discriminate: disagree	0.0006^***^	0.0057^***^	0.1051^***^	−0.0335^***^	−0.0782^***^
Discriminate: agree to some extent	0.0014^***^	0.0123^***^	0.1901^***^	−0.0847^***^	−0.1191^***^
Discriminate: completely agree	0.0011^***^	0.0091^***^	0.1492^***^	−0.0583^***^	−0.1011^***^

* *p < 0.05*,

** *p < 0.01*,

*** *p < 0.001*.

From the perspective of other explanatory variables, for each additional unit of income, the probability of an individual feeling very happy is 0.0072 higher than the probability of being happy. Therefore, the higher the income, the stronger the individual's happiness. The happiness level of women is significantly higher than that of men. Individuals who choose to move outside the province and outside the city have a higher sense of happiness than those who move within the city. The longer the flow time, the higher the individual's happiness. The stronger the love of the city, the higher the individual's sense of happiness, on the contrary, the stronger the discrimination, the weaker the sense of happiness.

## Conclusion

In this study, we examined the relationship between education, income, and happiness among migrants in China. We found that education had a positive effect on income and happiness, and that it can directly affect individual happiness. Furthermore, it can also influence happiness via the mediating effect of income.

The results of this study provide some practical implications. First, the findings are different from those of empirical research by scholars on the contradictory relationship between education and happiness in the UK and European countries: the higher the level of education that China's individuals receive, the stronger their happiness level, indicating that in China, higher education can fulfill the role of enhancing happiness. Second, research has found that the income level can increase with the education level. This means that increasing investment in education can not only bring higher economic income to individuals, but also increase the per capita national income of the country. Third, there is an obvious positive correlation between income and happiness, and the happiness that education brings to individuals via the income mechanism is significant. This conclusion strongly refutes “Easterlin Paradox.” Therefore, individuals are encouraged to accumulate human capital by obtaining higher-level education, improving their academic qualifications and thus their salary earned, and therefore enhancing their direct happiness. Meanwhile, government should also seek to strengthen resource input into higher education, reduce the cost of education investment, improve the price mechanism of human capital and labor market, increase the rate of return from education, and achieve a simultaneous increase in income and happiness.

## Data Availability Statement

The raw data supporting the conclusions of this article will be made available by the authors, without undue reservation.

## Author Contributions

DY conceived and designed the research, provided guidance throughout the entire research process, and responsible for all R&R works. GZ and ML participated in data analysis and wrote and supplemented the English paper. HW reviewed and edited the article. All authors contributed to the article and approved the submitted version.

## Funding

The authors acknowledge funding support from the Major Program Project of the National Social Science Fund of China (No: 21AJL006) and the Key Program Project Ministry of Education of China (No: 16JJD790013).

## Conflict of Interest

The authors declare that the research was conducted in the absence of any commercial or financial relationships that could be construed as a potential conflict of interest.

## Publisher's Note

All claims expressed in this article are solely those of the authors and do not necessarily represent those of their affiliated organizations, or those of the publisher, the editors and the reviewers. Any product that may be evaluated in this article, or claim that may be made by its manufacturer, is not guaranteed or endorsed by the publisher.

## References

[1] Helliwell J Layard R Sachs JD De Neve J-E editors. World Happiness Report 2021: Happiness, Trust, Deaths Under COVID-19. (2021). Available online at: https://worldhappiness.report/ed/2021/ (accessed December 28, 2021).

[2] Urry HL Nitschke JB Dolski I Jackson DC Dalton KM Mueller CJ. Making a life worth living: neural correlates of well-being. Psychol Sci. (2004) 15:367–72. 10.1111/j.0956-7976.2004.00686.x 15147488

[3] Diener E. Subjective well-being. Psychol Bull. (1984) 95:542–75. 10.1037/0033-2909.95.3.542 6399758

[4] Kringelbach ML Berridge KC. Towards a functional neuroanatomy of pleasure and happiness. Trends Cogn Sci. (2009) 13:479–87. 10.1016/j.tics.2009.08.006 19782634 PMC2767390

[5] Andrews FM Stephen B. Withey Social Indicators of Well-Being: Americans' Perceptions of Life Quality. New York, NY: Plenum Press (1976).

[6] Zhang Q Yang Y Zhang GL. Influence of life meaning on subjective well-being of older people: serial multiple mediation of exercise identification and amount of exercise. Front Public Health. (2021) 9:515484. 10.3389/fpubh.2021.515484 34307265 PMC8295607

[7] Veenhoven R. Happy life-expectancy, a comprehensive measure of quality of life in nations. Soc Indic Res. (1996) 39:1–58. 10.1007/BF00300831

[8] Bentham J. An Introduction to the Principle of Morals and Legislation. New York, NY: Methuen & Co Ltd. (1982).

[9] Jevons WS. The Theory of Political Economy. London: Macmillan (1871).

[10] Noddings N. Happiness and Education. Cambridge: Cambridge University Press (2003).

[11] Roberts P. Happiness, despair and education. Stud Philos Educ. (2013) 32:463–75. 10.1007/s11217-012-9325-427123227

[12] Nikolaev B Rusakov P. Education and happiness: an alternative hypothesis. Appl Econ Lett. (2016) 23:827–30. 10.1080/13504851.2015.1111982

[13] Tan H Luo J Zhang M. Higher education, happiness, and residents' health. Front Psychol. (2020) 11:1669. 10.3389/fpsyg.2020.01669 32849017 PMC7396533

[14] Veenhoven R. Healthy happiness: effects of happiness on physical health and the consequences for preventive health care. J Happiness Stud. (2008) 9:449–69. 10.1007/s10902-006-9042-1

[15] Cunado J de Gracia FP. Does education affect happiness? Evidence for Spain. Soc Indic Res. (2012) 108:185–96. 10.1007/s11205-011-9874-x

[16] Moussa NM Ali WF. Exploring the relationship between students' academic success and happiness levels in the higher education settings during the lockdown period of COVID-19. Psychol Rep. (2021) 33:294121994568–33294121994568. 10.1177/0033294121994568 33573504 PMC9006096

[17] Nikolaev B. Does higher education increase hedonic and eudaimonic happiness? J Happiness Stud. (2018) 19:483–504. 10.1007/s10902-016-9833-y33995163

[18] Sheldon KM Kasser T. Pursuing personal goals: skills enable progress, but not all progress is beneficial. Pers Soc Psychol Bull. (1998) 24:1319–31. 10.1177/01461672982412006

[19] Brighouse H. On Education. New York, NY: Routledge (2006).

[20] Michalos AC. Education, happiness and wellbeing. Soc Indic Res. (2008) 87:347–66. 10.1007/s11205-007-9144-0

[21] Schultz TW. Capital formation by education. J Polit Econ. (1960) 68:571–83. 10.1086/258393

[22] Spence M. Job market signaling. Q J Econ. (1973) 87:355–74. 10.2307/1882010

[23] Weiss A. Human capital vs. signalling explanation of wages. J Econ Perspect. (1995) 9:133–54. 10.1257/jep.9.4.133

[24] Mincer J. Schooling, Experience and Earnings. New York, NY: Columbia University Press (1974).

[25] Moen ER. Education, ranking, and competition for jobs. J Labor Econ. (1999) 17:694–723. 10.1086/20993634551023

[26] Blanchflower DG Oswald AJ. Well-being over time in Britain and the USA. J Public Econ. (2004) 88:1359–86. 10.1016/S0047-2727(02)00168-8

[27] Samuelson PA. A note on the pure theory of consumer's behavior. Economica. (1938) 5:353–4. 10.2307/2548836

[28] Tran DB Pham TDN Nguyen TT. The influence of education on women's well-being: evidence from Australia. PLoS ONE. (2021) 16:1–15. 10.1371/journal.pone.0247765 33760853 PMC7990187

[29] Piao X Ma X Managi S. Impact of the intra-household education gap on wives' and husbands' well-being: evidence from mross-country microdata. Soc Indic Res. (2021) 156:111–36. 10.1007/s11205-021-02651-5

[30] Ruiu G Ruiu ML. The complex relationship between education and happiness: the case of highly educated individuals in Italy. J Happiness Stud. (2019) 20:2631–53. 10.1007/s10902-018-0062-4

[31] Clark AE Oswald AJ. Satisfaction and comparison income. J Public Econ. (1996) 61:359–81. 10.1016/0047-2727(95)01564-7

[32] Wilson WR. Correlates of avowed happiness. Psychol Bull. (1967) 67:294–306. 10.1037/h0024431 6042458

[33] Jongbloed J. Higher education for happiness? Investigating the impact of education on the hedonic and eudaimonic well-being of Europeans. Eur Educ Res J. (2018) 17:733–54. 10.1177/1474904118770818

[34] Easterlin RA McVey LA Switek M Sawangfa O Zweig JS. The happiness—income paradox revisited. PNAS. (2010) 107:22463–8. 10.1073/pnas.1015962107 21149705 PMC3012515

[35] FitzRoy FR Nolan MA. Education, income and happiness: panel evidence for the UK. Empir Econ. (2020) 58:2573–92. 10.1007/s00181-018-1586-5

[36] Doh YY Chung J. What types of happiness do Korean adults pursue?—Comparison of seven happiness types. Int J Environ Res Public Health. (2020) 17:1502. 10.3390/ijerph17051502 32110951 PMC7084433

[37] Chen N Chen H Lin S. Effect of education–occupation mismatch on happiness. Int J Soc Econ. (2020) 47:86–110. 10.1108/IJSE-04-2019-0283

[38] Araki S. Correction to: does education make people happy? Spotlighting the overlooked societal condition. J Happiness Stud. (2021) 23:631–3. 10.1134/S2079057021010471

[39] Yang J Liu K Zhang Y. Happiness inequality in China. J Happiness Stud. (2019) 20:2747–71. 10.1007/s10902-018-0067-z

[40] Zhang Y Liu H. Individual's gender ideology and happiness in China. Chin Soc Rev. (2021) 53:1–26. 10.1080/21620555.2021.1871727 PMC9268205 35814530

[41] Xiao JJ Yan C Bialowolski P Porto N. Consumer debt holding, income and happiness: evidence from China. Int J Bank Mark. (2021) 39:789–809. 10.1108/IJBM-08-2020-0422

[42] Wang P Pan J Luo Z. The impact of income inequality on individual happiness: evidence from China. Soc Ind Res. (2015) 121:413–35. 10.1007/s11205-014-0651-5

[43] Bonnefond C Mabrouk F. Subjective well-being in China: direct and indirect effects of rural-to-urban migrant status. Rev Soc Econ. (2019) 77:442–68. 10.1080/00346764.2019.1602278

[44] Baron RM Kenny DA. The moderator-mediator variable distinction in social psychological research: conceptual, strategic, and statistical considerations. J Pers Soc Psychol. (1986) 51:1173–82. 10.1037/0022-3514.51.6.1173 3806354

[45] Judd CM Kenny DA. Process analysis: estimating mediation in treatment evaluations. Eval Rev. (1981) 5:602–19. 10.1177/0193841X8100500502

[46] Sobel ME. Direct and indirect effects in linear structural equation models. Socio Meth and Res. (1987) 16:155–76. 10.1177/004912418701600100626648886

[47] Becker GS. Investment in human capital: a theoretical analysis. J Polit Econ. (1962) 70:9–49. 10.1086/258724

[48] Ross CE Willigen MV. Education and the subjective quality of life. J Health Soc Behav. (1997) 38:275–97. 10.2307/2955371 9343965

[49] Chen WC. How education enhances happiness:comparison of mediating factors in four East Asian countries. Soc Indic Res. (2012) 106:117–31. 10.1007/s11205-011-9798-5

[50] Layard R. Rethinking public economics: the implications of rivalry and habit. In: Bruni, Porta, editors. Economies and Happiness: Framing the Analysis. Oxford: Oxford University Press (2005). p. 147–69. 10.1093/0199286280.003.0006

[51] Pega F Náfrádi B Momen NC Ujita Y Streicher KN Prüss-Üstün AM. Global, regional, and national burdens of ischemic heart disease and stroke attributable to exposure to long working hours for 194 countries, 2000–2016: a systematic analysis from the WHO/ILO joint estimates of the work-related burden of disease and injury. Environ Int. (2021) 154:106595. 10.1016/j.envint.2021.106595 34011457 PMC8204267

